# Prevalence of and risk factors for adolescent scoliosis from a multi-year school screening programme in Eastern China

**DOI:** 10.3389/fped.2025.1524073

**Published:** 2025-06-04

**Authors:** Rong Xu, Jianghui Li, Weihong Wang, Lijun Zhang, Hua Liu

**Affiliations:** ^1^ Department of Pediatric Surgery, Kunshan Maternity and Children’s Health Care Hospital, Kunshan, China; ^2^ Department of Rehabilitation, Kunshan Disabled Persons Federation, Kunshan, China; ^3^ Department of Neurosurgery, Affiliated Kunshan Hospital of Jiangsu University, Kunshan, China

**Keywords:** scoliosis, school screening, prevalence, associated factors, adolescent

## Abstract

**Objective:**

This study aimed to determine the occurrence of adolescent scoliosis (AS) and identify possible associated factors in Eastern China.

**Methods:**

The screening technique involved performing forward bending tests and using scoliometer data. Adolescents at risk for scoliosis based on the screening were advised to undergo an x-ray examination for diagnosis confirmation.

**Results:**

Between 2019 and 2023, a total of 90,635 adolescents, comprising 41,836 females and 48,799 males, aged 11–18, underwent screening. Among the screened adolescents in Eastern China, the overall prevalence of scoliosis was 0.62%, with 0.99% in females and 0.30% in males. Independently associated factors were identified as female gender (OR = 1.319, 95% CI 1.031–1.686, *P* = 0.027), BMI ≤ 20 (OR = 2.959, 95% CI 2.271–3.855, *P* < 0.001), a tendency to incline towards one side (OR = 2.129, 95% CI 1.564–2.898, *P* < 0.001), and a habit of bending over the desk (OR = 1.523, 95% CI 1.079–2.150, *P* = 0.017).

**Conclusion:**

The current study found that the occurrence rate of AS in Eastern China is 0.62%. Female adolescents who are thin and tall and have poor learning posture are more susceptible to developing scoliosis.

## Introduction

Adolescent scoliosis (AS) is the most prevalent type of spinal deformity, affecting 1%–4% of the population (1, 2). Due to the lack of symptoms in the initial phases of the disease, AS is often ignored by patients and their caregivers, leading to a delay in its identification and treatment. AS usually appears during adolescence, specifically between the ages of 11 and 18, and accounts for most cases of scoliosis (3). In contrast to obesity and myopia, which are more easily recognized among school-aged children and have a higher prevalence, scoliosis has garnered limited attention (4). It is important to emphasize that scoliosis can advance rapidly and possibly result in a debilitating condition if not promptly identified and treated (5).

Scoliosis screening plays a crucial role in the early detection of scoliosis, enabling the identification of patients at an early stage when intervention is most effective. Early identification through screening facilitates the prompt initiation of conservative treatments, including observation, exercise, bracing, and, in more severe cases, surgical correction. These interventions are designed to halt the progression of spinal curvature and prevent long-term health complications, such as pulmonary disorders, disabilities, back pain, cosmetic concerns, and a diminished quality of life (2). By intervening early, scoliosis can be managed in ways that reduce the need for more invasive treatments and improve overall outcomes for patients. Furthermore, historical studies, such as the work by Grivas, have demonstrated that features like rib and vertebral asymmetries are often secondary rather than primitive, further underscoring the importance of early screening. These findings support the idea that proactive, early intervention through scoliosis screening is vital for preventing the condition from escalating, ensuring better patient outcomes and reducing the overall burden of scoliosis.

The establishment of China's National Monitoring Program for Common Diseases and Health Influencing Factors Among Students in 2018 aimed to enhance the early diagnosis of diseases in children and adolescents. Since the beginning of this program, specific schools have been regularly monitored for common health conditions, such as scoliosis, at defined intervals (6). This monitoring program and similar strategies have allowed for recent reports regarding scoliosis screening data among school-aged children in Mainland China (7). There is a lack of epidemiological data on scoliosis in Kunshan, the most affluent city at the county level in China. Hence, the primary objective of this 5-year scoliosis screening study was to detect children with scoliosis in this specific area and obtain a deeper understanding of the correlation between its occurrence and other causes.

## Materials and methods

### Ethical approval

This cross-sectional study, conducted over five years, aimed to assess second-grade junior high school students (8th grade) in Kunshan, Jiangsu, ChinaThe Institutional Review Board of Affiliated Kunshan Hospital of Jiangsu University approved this study. All methods were conducted in accordance with relevant institutional guidelines and ethical regulations in effect at the time of the study. All steps were carried out according to relevant instructions and regulations. Adolescents and their parents received information about the screening process. Parents or guardians of the children provided their written informed consent.

### Screening and inclusion criteria

All eighth-grade students from all 46 junior high schools were included without any form of exclusion or filtering. Scoliosis screening was performed by well-trained physicians using the Adams forward-bending test (8), and the angle of trunk rotation (ATR) was measured by a scoliometer (Orthopedic Systems, Inc., CA, USA). Adolescents who tested positive, with an ATR greater than 5°, were formally referred to the hospital for radiographic evaluation and confirmation of diagnosis.Each child was only examined once during the screening process.

### Collected variables

In this study, several key variables were collected for each participant. Demographic information such as age (in years) and gender (male or female) were recorded. Body Mass Index (BMI) was calculated based on participants' weight and height (kg/m²). To categorize BMI, adolescents were classified as BMI ≤ 20 kg/m² (indicating underweight or normal weight) and BMI > 20 kg/m² (indicating overweight or obesity). Additionally, information regarding postural habits was collected. This included whether participants had a tendency to incline towards one side (assessed during the forward-bending test or based on self-reported sitting habits) and whether they bent over the desk (assessed through self-reports or classroom observations). Other postural habits were also considered, such as backpack carrying (whether carrying one backpack ≤5 kg or two backpacks >5 kg), crossing legs, sitting with a bent waist, and incorrect pen grip posture, all of which were noted as either present or absent during the screening process.

### Data preprocessing

Once the data were collected, they were entered into an electronic database for analysis. A number of preprocessing steps were applied to ensure the quality and accuracy of the data. Any **missing data** or incomplete entries were excluded from the analysis to prevent bias, and no data imputation was performed. Extreme values, or **outliers**, were detected for continuous variables such as age, height, and weight, and any data points more than three standard deviations from the mean were excluded from the analysis. Additionally, continuous variables like BMI were categorized into predefined groups, as mentioned above, to facilitate statistical analysis. These steps ensured that the dataset was clean and ready for reliable statistical evaluation.

### Referral process

Once students were identified with a potential scoliosis diagnosis based on the forward-bending test and ATR measurement, a formal referral process was initiated. Those with ATR angles exceeding 5° were referred to the Department of Pediatric Orthopedics at Affiliated Kunshan Hospital of Jiangsu University for further evaluation. The referral was communicated through an electronic health record system, which allowed for the secure transfer of relevant patient data (including test results, demographic information, and preliminary assessments). Parents or guardians were provided with a referral letter, and transportation assistance was offered when needed. At the hospital, a more thorough examination was performed using x-ray imaging to confirm the diagnosis. This process ensured timely and appropriate treatment options for the adolescents.

### Data collection

Data collection occurred in two phases: initial screening at schools and follow-up evaluation at hospitals. All relevant information, including demographic data and diagnostic results, was entered into a secure electronic database. Strict confidentiality and ethical standards were maintained throughout the process, and access to data was limited to authorized personnel.

### Statistical analysis

All statistical analyses were performed utilizing SPSS version 29.0 (Chicago, IL, USA). The data were presented as mean ± standard deviation for continuous variables. For categorical data, counts and percentages were displayed. The continuous variables were compared using independent sample t-tests, while the categorical variables were compared using Pearson chi-squared tests. *P* values of <0.05 were considered statistically significant. Logistic regression analysis was performed to explore the factors associated with scoliosis. Potential factors were selected through the use of univariate logistic analysis. The multivariate logistic analysis included significant components to identify independent factors linked with scoliosis. The odds ratio (OR) and 95% confidence interval (CI) were presented.

## Results

### Summary of screening

From October 2019 to May 2023, 90,635 adolescents aged 11–16 years were screened, including 41,836 females (46.2%) and 48,799 males (53.8%). Most screened children were 13–14 years old (99.1%) (Table 1). The eleven districts and towns in Kunshan were home to all the adolescents who underwent screening; the highest percentage came from the High-tech district (32.6%), while the lowest percentage came from Zhouzhuang town (0.8%).

**Table 1:** Demographic characteristics of the study population included in the 5-year scoliosis screening program (*n* = 90,635).

Variable	Number
Gender	
Boys	48,799 (53.8)
Girls	41,836 (46.2)
Age	
All	13.51 ± 0.520
11	13 (0.0)
12	109 (0.1)
13	45,197 (49.9)
14	44,615 (49.2)
15	669 (0.7)
16	32 (0.0)
District	
Bacheng town	5,746 (6.3)
Development district	19,534 (21.6)
Dianshan Lake town	1,455 (1.6)
High-tech district	29,520 (32.6)
Huaqiao town	7,946 (8.8)
Jinxi town	1,034 (1.1)
Lujia town	5,889 (6.5)
Qiandeng town	4,773 (5.3)
Zhangpu town	4,944 (5.5)
Zhouzhuang town	743 (0.8)
Zhoushi town	9,051 (10.0)

Data are number (%) or means ± SD.

### Screening results by gender

**Figure 1:**
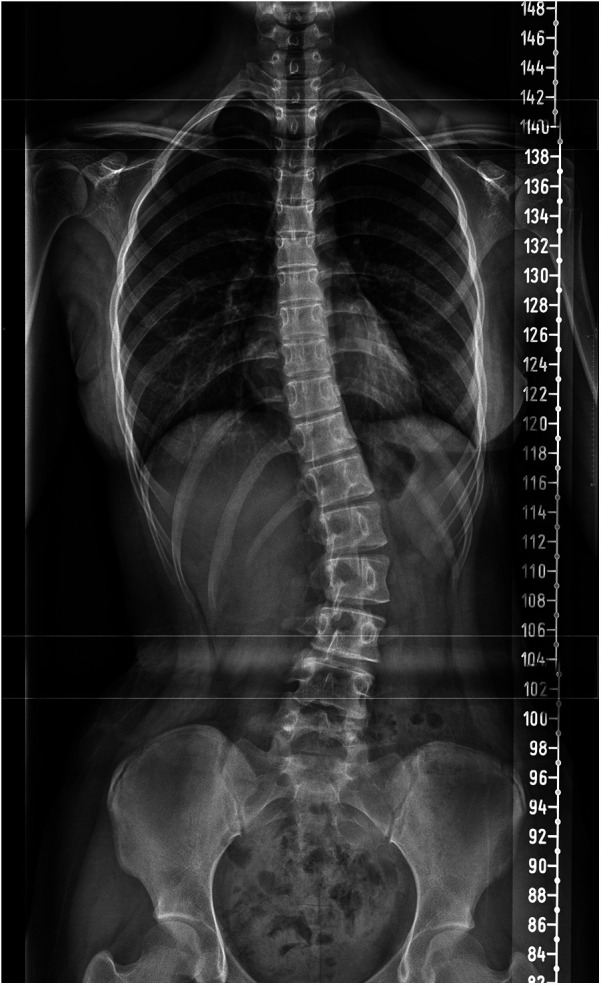
Representative spinal x-ray image of a 16-year-old girl diagnosed with adolescent scoliosis.

During the phase of school screening, 10,103 (4,915 males and 5,188 females) out of 90,635 (11.1%) adolescents were found to be positive for scoliosis and thus required further radiographic evaluation (Table 2). The positive rates of boys and girls were 10.1% (4,915/48,799) and 12.4% (5,188/41,836), respectively, and the difference was statistically significant (*χ*^2^ = 123.348, *P* < 0.001). Out of the individuals who tested positive, 14.4% (1,458 out of 10,103) went to the local hospitals to undergo x-ray diagnosis. Out of the children who underwent radiographic testing, a total of 559 individuals (144 males and 415 females) were conclusively diagnosed with scoliosis (Figure 1). This prevalence translates to only 0.62% (559/90,635) of the total population screened. Of these, 506 cases (90.5%, 506/559) had mild scoliosis (Cobb angle 10–25°), and 53 cases (9.5%, 53/559) showed moderate scoliosis (Cobb angle 25–40°).

**Table 2:** Screening outcomes by gender among adolescents over a 5-year period in Eastern China.

Variable	Boy	Girl	Total	Chi-Square	*P*-value
Total screen	48,799	41,836	90,635	123.348	<0.001
Negative	43,884 (89.9)	36,648 (87.6)	80,532		
Positive	4,915 (10.1)	5,188 (12.4)	10,103		
Performed radiography	537 (36.8)	921 (63.2)	1,458	6.537	0.01
Positive imaging	144	415	559	12.831	<0.001
10 ≤ Cobb < 25	116 (80.6%)	390 (94.0%)	506		
25 ≤ Cobb < 40	28 (19.4%)	25 (6.0%)	53		

The values in parentheses are percentages unless indicated otherwise.

### Yearly screening results

Next, the screening results were calculated for each scanning period (Table 3). The results indicated a gradual annual increase in the number of screenings. While there was a statistically significant variation in the percentage of anomalies detected each year, it remained rather stable at around 11%. Furthermore, although the annual number of teenagers referred to local hospitals for imaging operations was relatively small, significant discrepancies have been observed among them (Table 3). There was no significant difference in the proportion of diagnosed cases of scoliosis annually.

**Table 3:** Annual results of adolescent scoliosis screening over five consecutive years (2019–2023).

Screening period	2019	2020	2021	2022	2023	Chi-Square	*P* value
Total screen						88.779	<0.001
Negative	12,827 (87.6)	14,330 (87.4)	16,382 (89.6)	17,709 (89.8)	19,284 (89.3)		
Positive	1,817 (12.4)	2,060 (12.6)	1,905 (10.4)	2,015 (10.2)	2,306 (10.7)		
Performed radiography	326	208	368	403	153	447.672	<0.001
Positive imaging						5.313	0.257
10 ≤ Cobb < 25	121 (91.7)	76 (93.8)	128 (90.8)	144 (90.6)	37 (80.4)		
25 ≤ Cobb < 40	11 (8.3)	5 (6.2)	13 (9.2)	15 (9.4)	9 (19.6)		

The values in parentheses are percentages unless indicated otherwise.

### Characteristics with positive imaging

Subsequently, an analysis was conducted to examine the demographic characteristics of adolescents who had positive imaging (Table 4). These data showed that the prevalence in females (74.2%, 415/559) was significantly higher than in males (25.8%, 144/559) (*P* < 0.001). Additionally, the two groups had statistically considerably different body mass index (BMI) (*P* < 0.001). Furthermore, a significant association was found between the prevalent improper postural behaviors of adolescents while studying, such as leaning to one side and bending over the desk, and a favorable result in imaging tests.

**Table 4:** Comparison of demographic and postural characteristics between adolescents with and without radiographically confirmed scoliosis.

Variable	Negative Imaging	Positive Imaging	*P*-value*
Gender			<0.001
Boy	312 (32.9)	144 (25.8)	
Girl	587 (65.3)	415 (74.2)	
Back pack carrying			0.393
1	228 (25.4)	133 (23.8)	
2	671 (74.6)	426 (76.2)	
BMI			<0.001
≤20 kg/m²	554 (61.6)	462 (82.8)	
>20 kg/m²	345 (38.4)	96 (17.2)	
Incline towards one side			<0.001
No	799 (88.9)	451 (80.7)	
Yes	100 (11.1)	108 (19.3)	
Bend over the desk			0.019
No	816 (90.8)	485 (86.8)	
Yes	83 (9.2)	74 (13.2)	
Cross the legs			0.149
No	773 (86.0)	496 (88.7)	
Yes	126 (14.0)	63 (11.3)	
Sit with a bent waist			0.097
No	736 (81.9)	477 (85.3)	
Yes	163 (18.1)	82 (14.7)	
Incorrect pen grip posture			0.236
No	726 (80.8)	466 (83.4)	
Yes	173(19.2)	93(16.6)	

The values in parentheses are percentages unless indicated otherwise.

* *χ*^2^ test with Yates' correction.

### Univariate logistic regression analysis

Several characteristics significantly correlated with scoliosis using univariate logistic regression analysis (Table 5). A substantial correlation was found for gender, with an OR of 1.532 (95% CI 1.212–1.936, *P* < 0.001). Similarly, BMI demonstrated a strong association, with an OR of 3.003 (95% CI 2.321–3.886, *P* < 0.001). Additionally, inclining towards one side exhibited a significant association (OR = 1.913, 95% CI 1.424–2.571, *P* < 0.001), as did bending over the desk (OR = 1.500, 95% CI 1.075–2.093, *P* = 0.017). These results highlight that the etiology of scoliosis is multifaceted and involves both physiological and environmental components.

**Table 5:** Univariate and multivariate logistic regression analysis of factors associated with radiographically confirmed scoliosis.

Variables	Univariate Analysis			Multivariate Analysis		
	*P*	OR	95% CI	*P*	OR	95% CI
Gender	<0.001			0.027		
Male		Ref	-		Ref	-
Female		1.532	1.212–1.936		1.319	1.031–1.686
Back pack carrying	0.500					
1		Ref	-			
2		1.088	0.851–1.392			
BMI	<0.001			<0.001		
≤20 kg/m^2^		3.003	2.321–3.886		2.959	2.271–3.855
>20 kg/m^2^		Ref	-		Ref	-
Incline towards one side	<0.001			<0.001		
No		Ref	-		Ref	-
Yes		1.913	1.424–2.571		2.129	1.564–2.898
Bend over the desk	0.017			0.017		
No		Ref	-		Ref	-
Yes		1.500	1.075–2.093		1.523	1.079–2.150
Cross the legs	0.130					
No		Ref	-			
Yes		1.283	0.929–1.772			
Sit with a bent waist	0.086					
No		Ref	-			
Yes		1.288	0.965–1.720			
Incorrect pen grip posture	0.211					
No		Ref	-			
Yes		1.194	0.905–1.576			

### Multivariate logistic regression analysis

**Figure 2:**
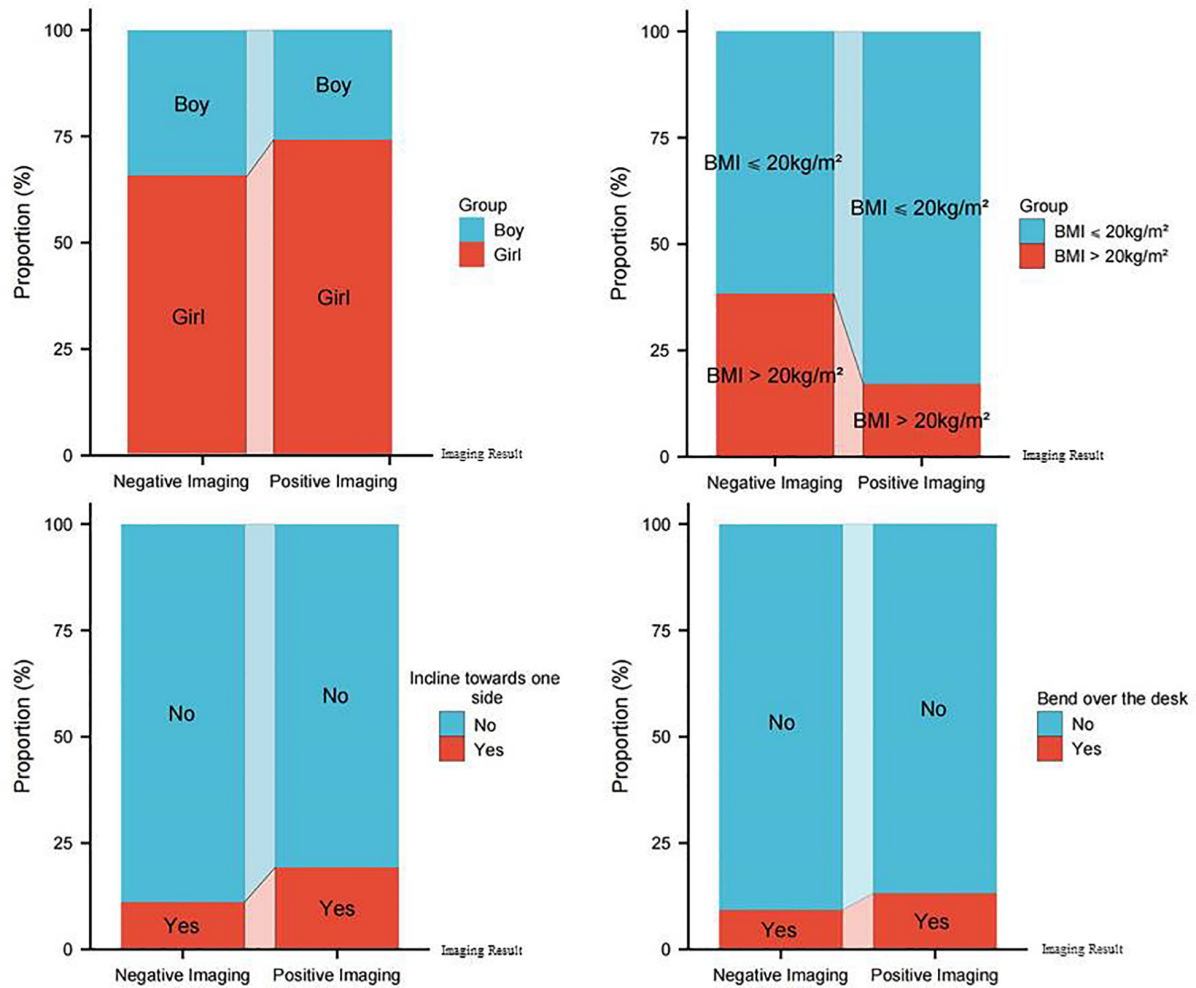
Comparison of demographic and postural characteristics between adolescents with positive and negative scoliosis imaging results.

In multivariate logistic regression analysis, several independent factors were identified as associated with scoliosis (Table 5). Female gender exhibited a significant association, with an OR of 1.319 (95% CI 1.031–1.686, *P* = 0.027). Additionally, individuals with a BMI ≤ 20 demonstrated a strong association with scoliosis, with an OR of 2.959 (95% CI 2.271–3.855, *P* < 0.001). Inclining towards one side also showed a significant association (OR = 2.129, 95% CI 1.564–2.898, *P* < 0.001), as did bending over the desk (OR = 1.523, 95% CI 1.079–2.150, *P* = 0.017). These results demonstrate the separate roles that gender, BMI, and certain postural habits play in developing scoliosis. The overlay diagram illustrates how the positive imaging group had a considerably higher proportion of females with BMI ≤ 20, bend over the desk, and inclined rewards on one side (Figure 2).

## Discussion

The present study lasted five years, during which we conducted screenings for scoliosis among adolescents in school. Early identification of scoliosis is crucial for effectively controlling the condition and preventing its further advancement. Throughout the study, many children underwent screening, which involved physical examination and radiographic imaging. This allowed for the collection of extensive data on each adolescent.

In this study, the prevalence of scoliosis was 0.62%, comparable to the rates in Japan (0.87%), Singapore (0.59%) and India (0.61%), yet lower than those in Turkey (2.30%), Greece (1.70%), Brazil (1.50%) and Nigeria (1.20%) (9–15). In China, the prevalence of scoliosis varies significantly across different regions, ranging from 0.11%–3.69% (1, 2, 6, 7). The analysis revealed that the rare prevalence of scoliosis in Kunshan can be ascribed to its economic prosperity as a constituent of the Yangtze River Delta. Hence, it is practical and convenient to refer suspected cases of scoliosis to Shanghai hospitals, which provide a wider range of medical services. This could potentially account for the higher screening positive rate we have observed (11.1%, 10,103/90,635) compared to Shanghai (6.9%) (6) and the lower diagnosis rate of scoliosis (0.62% vs. 2.00%) (2).

It is worth noting that although the positive screening rate was higher in 2019 than in 2021 and 2022, the number of patients sent to local hospitals for further assessment was lower than in 2021 and 2022 but higher than in 2020 and 2023 (Table 3). One potential explanation for this finding is that during the years 2020 and 2023, adolescents who were feeling positive had unhindered entry to the main hospitals in Shanghai to receive medical care. Referrals were restricted during the COVID-19 pandemic from 2021–2022. As a result, the prevalence documented in this study could potentially be underestimated. The claimed prevalence rates can be considerably influenced by the accessibility of medical treatment and the presence of scoliosis screening programs. Regions with superior healthcare infrastructure and a higher level of scoliosis education are more prone to effectively diagnosing and documenting cases of scoliosis.

Logistic regression analysis identified several factors associated with the occurrence of AS, with female gender being a significant predictor, consistent with earlier research (2, 16). Although females predominated in the study population, the gender difference in scoliosis prevalence is not fully understood. This discrepancy may be due to a combination of hormonal, anatomic, and biomechanical factors (16). For instance, females tend to have wider and flatter pelvises and more flexible spines, making them more susceptible to scoliosis. Additionally, females undergo puberty earlier, and the hormonal changes during this period may influence spinal development. Despite these factors, a notable difference between clinical rib deviation and radiological spinal deviation remains, particularly in younger adolescents. As highlighted by the work by Grivas and colleagues (2024) (17), this discrepancy underscores the challenge in correlating clinical signs, such as rib deformity, with radiologically confirmed spinal curvature. The fact that rib deformity does not always correspond directly to spinal curvature in younger individuals emphasizes the importance of early screening and radiographic evaluation to ensure accurate scoliosis diagnosis. Healthcare practitioners should be aware of gender disparities and the potential for discrepancies between clinical and radiological findings. Incorporating scoliosis screening into regular healthcare visits for adolescents can help identify scoliosis early, prevent its progression, and mitigate any long-term issues.BMI is a comprehensive indicator of body shape and nutritional status, which plays an important role in the development of various skeletal conditions, including scoliosis (8) In line with previous studies, such as those by Zhou et al. (1). (2022), which reported a lower BMI among children with scoliosis in the Qinghai-Tibetan Plateau, our study also found a significant association between low BMI and increased risk of adolescent scoliosis (AS) in the Eastern China population. Specifically, adolescents with a BMI ≤ 20 had a 2.959-fold higher prevalence of scoliosis compared to those with a BMI > 20, consistent with previous findings (18). The association between low BMI and scoliosis can be explained by several factors. First, adolescents with lower BMI generally have less adipose and muscle tissue surrounding the spine, which may reduce the natural support for spinal stability. As a result, their spines may become more susceptible to external stresses, increasing the likelihood of developing scoliosis. Furthermore, low body weight is often a marker of inadequate nutrition, which can impair bone development and contribute to fragile bone structures, making the spine more vulnerable to deformities. In addition to these physiological factors, low BMI may also influence the diagnostic process. Adolescents with a lower BMI tend to have more pronounced spinal curvature due to the reduced soft tissue around the spine, making scoliosis more visually apparent during screening. This could lead to a higher likelihood of being diagnosed with scoliosis based on physical appearance. It is also possible that low BMI reflects underlying factors such as malnutrition or genetic predispositions that contribute to both poor bone health and spinal deformities. Therefore, while low BMI may indeed be a risk factor for scoliosis, the association between these factors is likely influenced by both physical appearance and biological conditions, highlighting the complexity of this relationship.

The findings of this study highlighted the critical clinical and public health importance of early detection and intervention for adolescent scoliosis. Early identification through school-based screening programs is essential in preventing the progression of scoliosis, which can lead to long-term complications such as chronic pain, disability, and cosmetic concerns. By detecting scoliosis early, timely interventions like observation, exercise, or bracing can be implemented, reducing the need for more invasive treatments such as spinal surgery. Furthermore, our findings point to key risk factors such as gender, BMI, and posture-related habits, which can be addressed through targeted public health campaigns. Promoting proper posture, regular physical activity, and ergonomic habits in schools can significantly reduce the incidence of scoliosis. From a clinical perspective, healthcare providers should focus on high-risk groups, particularly females and those with low BMI, to ensure early diagnosis and effective management. On a broader public health scale, implementing routine scoliosis screenings in schools and raising awareness about preventive strategies can significantly reduce the healthcare burden and improve long-term health outcomes for adolescents.

This study revealed that prolonged sitting with an inclined posture towards one side and bending over the desk were significantly correlated with the onset of scoliosis among adolescents. The persistent stress and asymmetry on the spinal column caused by these postural behaviors may explain the correlation. When a person leans to one side while sitting, it exerts uneven pressure on the vertebrae, potentially leading to spinal curvature. Similarly, prolonged periods of bending over the desk place excessive stress on the lumbar region, contributing to the onset of scoliosis. Although this study identified a significant association between these postural habits and scoliosis, the relationship remains controversial. While some studies suggest that poor posture, including bending over a desk, may contribute to spinal curvature, the exact nature of this relationship remains unclear. The development of scoliosis likely involves a combination of genetic, environmental, and postural factors, making it crucial to consider these factors when interpreting the link between posture and scoliosis (19). Given the findings, it is important to advocate for appropriate sitting habits among adolescents. Parents and educators should encourage students to sit upright with their backs straight, avoid leaning to one side, and take regular breaks to prevent prolonged bending over the desk. Additionally, physical activities that strengthen the back muscles, such as yoga and swimming, can help reduce the risk of scoliosis and should be actively promoted.

By targeting these adjustable risk factors, it is anticipated that the occurrence of scoliosis among teenagers can be diminished. Subsequent investigations should focus on validating these results and discovering supplementary preventative strategies to alleviate the impact of this condition further. Our study further demonstrates the significance of regular scoliosis screening in schools. Frequent screenings will ensure that children with scoliosis are found early and sent to the right place for care. Thus, it may be possible to prevent the disease from becoming severe, resulting in more serious health issues.

## Limitations

This study has several limitations. First, while the large sample size improves the reliability of our findings, the study is based on adolescents from a specific region in Eastern China, limiting the generalizability of the results to other regions with different socioeconomic or healthcare contexts. The screening methodology—specifically the Adams forward-bending test and scoliometer measurements—may be influenced by examiner skill and interpretation, introducing potential bias in scoliosis detection. Although x-ray confirmation reduces this concern, examiner variability could still impact results. Additionally, self-reported data on postural habits may be subject to recall bias. A key limitation is the low rate of radiographic follow-up. Only 14.4% (1,458 out of 10,103) of positively screened individuals underwent x-ray confirmation, and 38.3% of them were diagnosed with scoliosis. Given that many adolescents with ATR >5° were not x-rayed, the actual prevalence could be higher than the reported 0.62%. Extrapolating the 38.3% confirmation rate to all screened individuals would yield an estimated prevalence of 4.3%, suggesting an underestimation of the true prevalence. Improving follow-up adherence in screening programs would help provide a more accurate prevalence estimate. Finally, the study's reliance on school-based screening may have led to selection bias, as adolescents less likely to participate in school activities might have been excluded. Future research should focus on enhancing referral compliance, using more objective posture assessments, and including more diverse populations.

## Conclusion

In conclusion, our findings indicate that the prevalence of scoliosis among children in Kunshan, Eastern China, is 0.62%. Our data suggest that female adolescents who are both thin and tall and have poor learning postures are at a higher risk of developing scoliosis. Comprehending these aspects is essential for formulating efficient scoliosis preventive and treatment programs customized to each location's specific requirements.

## Data Availability

The raw data supporting the conclusions of this article will be made available by the authors, without undue reservation.
